# Sex differences in insulin resistance and basal insulin secretion among elderly men and women without a history of diabetes: a comparative study between obese and non-obese groups

**DOI:** 10.1007/s13340-026-00904-7

**Published:** 2026-06-04

**Authors:** Hironari Sano, Yusaku Hayashi, Rimei Nishimura

**Affiliations:** 1 Division of Diabetes, Metabolism and Endocrinology, Department of Internal Medicine, Jikei University School of Medicine, 3-25-8, Nishishimbashi, Minato-ku, Tokyo 105-8461 Japan; 2 Division of Cardiology, Department of Internal Medicine, Jikei University School of Medicine, 3-25-8, Nishishimbashi, Minato-ku, Tokyo 105-8461 Japan; 3 Department of Internal Medicine, Tsunan Municipal Hospital, 2682, Shimofunatotei, Tsunan-machi, Nakauonuma-gun, Niigata 949-8201 Japan

**Keywords:** Early elderly, Late elderly, Obesity, Insulin resistance, Insulin secretion capacity

## Abstract

**Aim:**

The aim of this study was to examine sex differences in insulin resistance and basal insulin secretion among elderly individuals without a history of diabetes, comparing obese and non-obese groups in early elderly (aged 65–74 years) and late elderly (aged 75–89 years).

**Patients and methods:**

A total of 478 individuals (258 men and 220 women) who underwent comprehensive medical checkups at Tsunan Municipal Hospital between 2008 and 2014 were included. We investigated whether there were interactive effects between age and Body Mass Index (BMI) on homeostasis model assessment of insulin resistance (HOMA-IR) values and homeostasis model assessment of beta cell function (HOMA-β) values, and examined their associations with each factor using analysis of variance (ANOVA) and multivariate analysis.

**Results:**

In elderly men, elevated BMI was significantly associated with higher HOMA-IR values, and HOMA-β values were also significantly higher in the obese group than in the non-obese group. In contrast, among elderly women, HOMA-β values in the obese group were comparable to those in the non-obese group regardless of early and late elderly.

**Conclusions:**

In elderly men, insulin resistance associated with obesity was firmly present regardless of age, whereas, in elderly women, there is no difference in basal insulin secretion between the obese and non-obese groups irrespective of age.

## Introduction

The prevalence of impaired glucose metabolism has been reported to increase with advancing age [1]. The progression from impaired glucose tolerance to overt diabetes is influenced by both increased insulin resistance and a relative decline in insulin secretory capacity, with both factors serving as risk contributors [2], and these two factors are known to interact and mutually accelerate disease development [3]. Similar findings have become even more pronounced in elderly populations [4, 5]. According to the 2023 National Health and Nutrition Survey in Japan, the proportion of persons in whom diabetes is strongly suspected is highest among those aged 70 years and older, in both men and women [6]. Meanwhile, among individuals with normal glucose tolerance, it has been reported that men exhibit higher insulin resistance than women [7].

Focusing next on obesity, its prevalence has been continuously increasing both in Western countries [8] and in Japan [9]. Numerous studies from Western populations [10, 11] and Japan [12] have reported a close association between obesity and insulin resistance. However, few studies have examined in detail the relationship between obesity and insulin secretory capacity specifically in elderly individuals.

In this study, we focused on elderly individuals without a history of diabetes. Using the HOMA [13], we calculated HOMA-IR values as an index of insulin resistance and HOMA-β values as an index of basal insulin secretion. The aim was to compare HOMA-IR and HOMA-β values among early elderly (aged 65–74 years) and late elderly (aged 75–89 years), stratified by sex and by obesity status (obese and non-obese). We further investigated whether there were interactive effects between age and BMI on HOMA-IR values and HOMA-β values, and examined their associations with each factor using ANOVA and multivariate analysis.

### Subjects and methods

A total of 478 individuals (258 men and 220 women) who underwent comprehensive medical checkups at Tsunan Municipal Hospital between 2008 and 2014 were included in this study. Participants were eligible if they met all of the following criteria: (1) aged between 65 and 89 years, (2) HbA1c levels below 6.5%, and (3) no history of diabetes. At the time of the checkup, data were collected on sex, age, height, weight, fasting plasma glucose (FPG) levels, HbA1c levels, and fasting immunoreactive insulin (F-IRI) levels. Additional information was obtained regarding smoking status and the use of antihypertensive medications. HOMA-IR values and HOMA-β values were calculated using the HOMA [14].

Age, sample size, BMI, FPG levels, HbA1c levels, F-IRI levels, HOMA-IR values, HOMA-β values, smoking status, and the use of antihypertensive medications were stratified by total population, sex, age groups (early elderly: aged 65–74 years; late elderly: aged 75–89 years), and BMI groups (non-obese: BMI < 25; obese: BMI * \ge * 25). For age, BMI, FPG levels, HbA1c levels, F-IRI levels, HOMA-IR values and HOMA-β values as continuous variables, median values with interquartile ranges (25th–75th percentile) were expressed. For smoking status and the use of antihypertensive medications as categorical variables, counts and percentages were presented.

Comparisons were conducted between men and women, early and late elderly groups, and obese and non-obese groups. The Mann–Whitney U test was used to compare medians between two groups, while the chi-squared test and Fisher’s exact test were applied to compare proportions. A p-value of < 0.05 was considered statistically significant.

**Fig. 1:**
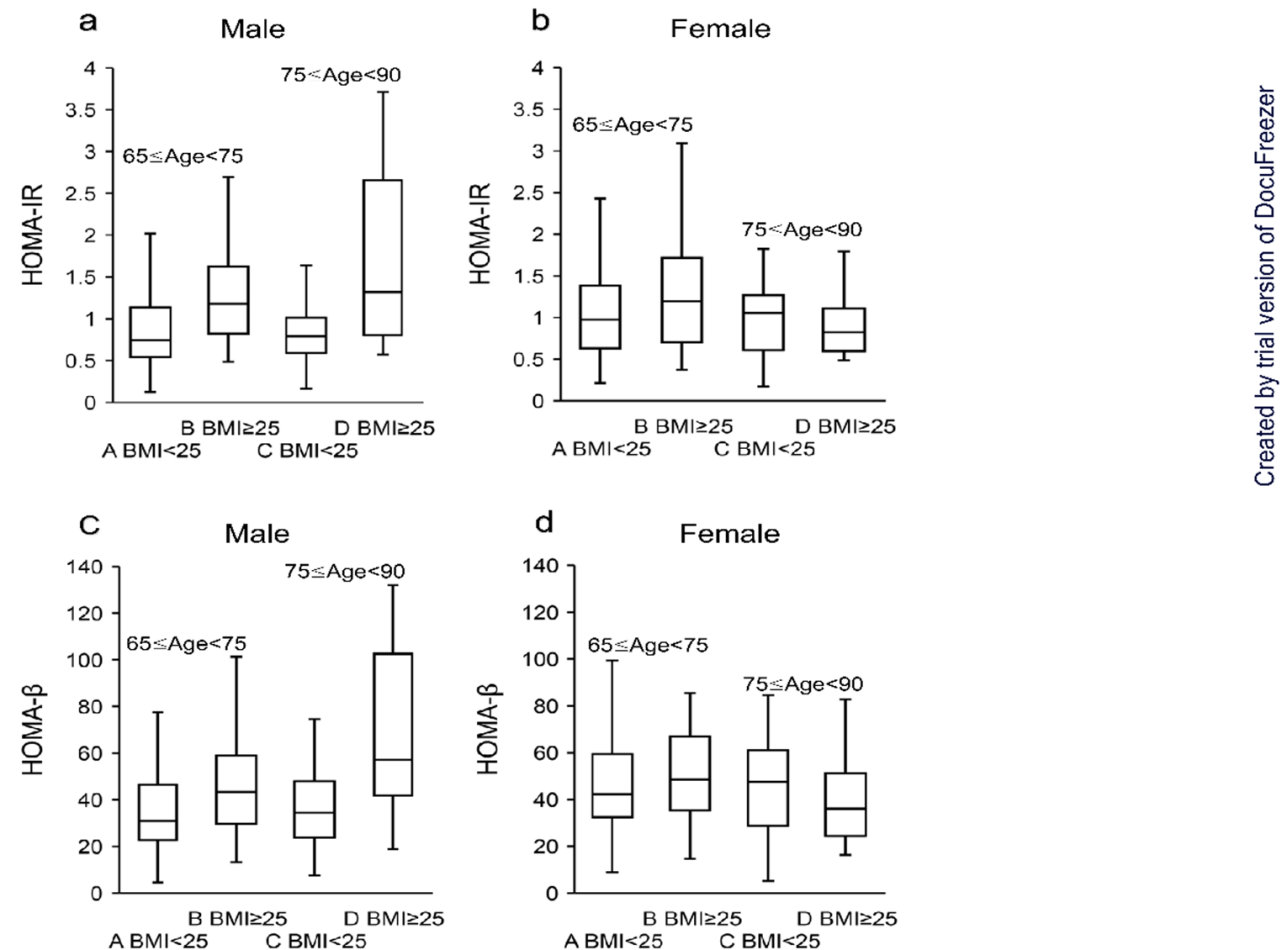
Distribution of HOMA-IR and HOMA-β values by sex across four groups. **a** Distribution of HOMA-IR values in male. **b** Distribution of HOMA-IR values in female. **c** Distribution of HOMA-β values in male. **d** Distribution of HOMA-β values in female. Group A: Non-obese individuals (BMI < 25) aged * \ge * 65 and < 75 years. Group B: Obese individuals (BMI * \ge * 25) aged * \ge * 65 and < 75 years. Group C: Non-obese individuals (BMI < 25) aged * \ge * 75 and < 90 years. Group D: Obese individuals (BMI * \ge * 25) aged * \ge * 75 and < 90 years. HOMA evaluation: HOMA-β (%) = F-IRI level (mU/mL) × 360 / FPG level (mg/dL) – 63 HOMA-IR = F-IRI level (mU/mL) × FPG level (mg/dL) / 405 These formulas reflect the evaluation methods for insulin secretion capability and insulin resistance used in the study.

Participants were stratified into two age groups (aged 65–74 years and aged 75–89 years) and two BMI groups (BMI * \ge * 25 and BMI < 25). To examine the effects of age, BMI, and their interaction on HOMA-IR values and HOMA-β values, a two-way ANOVA was conducted separately for men and women. The distributions of HOMA-IR and HOMA-β values were illustrated using box plots, stratified by sex, age group, and BMI category (Fig. 1). Furthermore, multiple comparison tests were performed to assess the simple main effects of age and BMI on HOMA-IR values and HOMA-β values, also stratified by sex. Both the two-way ANOVA and the multiple comparison tests were conducted using the Scheffé method. To examine factors associated with HOMA-IR values and HOMA-β values, multivariate analysis was performed using logistic regression, with HOMA-IR values and HOMA-β values as dependent variables. The independent variables included sex, age, BMI, the interaction between age and BMI, HbA1c levels, smoking status, and the use of antihypertensive medications. For the dependent variables, dummy coding was applied: HOMA-IR values * \ge * 2.5 were coded as 1 and < 2.5 as 0; HOMA-β values < 30 were coded as 1 and * \ge *   30 as 0. Dummy variables were also used for the independent variables: age 75–89 years was coded as 1 and age 65–74 years as 0; BMI * \ge * 25 as 1 and < 25 as 0; male as 1 and female as 0; smoking and the use of antihypertensive medications were coded as 1 for presence and 0 for absence. HbA1c levels were entered as a continuous variable, using values standardized by the National Glycohemoglobin Standardization Program (NGSP). All statistical analyses were conducted using Statistical Analysis System (SAS) version 9.4 [15].

The charting feature of Microsoft Excel was used to create the artwork.

## Results

### Comparison of factors by sex, age, and BMI

In males, compared to females, FPG levels and the proportion of smokers were significantly higher, whereas BMI, HbA1c levels, F-IRI levels, HOMA-β values and HOMA-IR values were significantly lower. No significant differences were observed across age groups. Among individuals with a BMI of 25 or higher, FPG levels, HbA1c levels, F-IRI levels, HOMA-β values and HOMA-IR values were significantly higher than those with a BMI below 25. The proportion of individuals taking antihypertensive medications was significantly higher in the BMI * \ge * 25 group compared to the BMI < 25 group (Table 1).

**Table 1:** Clinical characteristics-in overall and by sex, age and BMI

Age (years)	Overall	Male	Female	65 * łe * Age < 75	75 * łe * Age < 90	BMI < 25	25 * łe * BMI
	70 (67–74)	71 (67–74)	70 (67–75)	69 (67–72)	79**** (77–81)	70 (67–74)	71 (68–74)
N	478	258	220	366	112	372	106
Male (%)	258 (54%)	258 (100%)	0 (0%)	201 (55%)	57 (51%)	207 (56%)	51 (48%)
Female (%)	220 (46%)	0 (0%)	220 (100%)	165 (45%)	55 (49%)	165 (44%)	55 (52%)
BMI (kg/m^2^)	23.0 (21.2–24.8)	22.7 (21.1–24.5)	23.2* (21.4–25.1)	23.0 (21.4–24.7)	22.9 (20.7–24.9)	22.2 (20.7–23.5)	26.2**** (25.5–27.2)
FPG (mg/dl)	98 (92–104)	99 (93–105)	96*** (91–102)	98 (92–104)	97 (92–103)	97 (92–103)	100** (95–106)
HbA1c (%)	5.8 (5.6–6.0)	5.8 (5.5–5.9)	5.8* (5.6–6.0)	5.8 (5.6–6.0)	5.8 (5.6–6.0)	5.8 (5.6–5.9)	5.9* (5.7–6.0)
F-IRI	3.7 (2.5–5.2)	3.3 (2.4–5.0)	4.1** (2.8–5.6)	3.6 (2.5–5.3)	3.8 (2.6–5.1)	3.5 (2.4–5.0)	4.6**** (3.0–6.3)
HOMA-IR	0.88 (0.60–1.32)	0.82 (0.58–1.23)	0.98* (0.64–1.37)	0.88 (0.59–1.37)	0.87 (0.61–1.25)	0.83 (0.57–1.20)	1.11**** (0.70–1.62)
HOMA-β	39.3 (25.8–54.6)	34.9 (23.3–49.1)	42.9**** (32.4–60.2)	39.7 (26.5–54.9)	38.4 (25.1–53.4)	37.7 (25.4–52.1)	45.4*** (32.0–62.5)
Smoker	39 (8)	34 (13)	5 (2) ****	32 (9)	7 (6)	34 (9)	5 (5)
Antihypertensive agents	214 (45)	105 (41)	109 (50)	166 (45)	48 (43)	152 (41)	62 (58) **

Data are expressed as median (25–75%) and n(%), differences between male and female, early elderly group and late elderly group, non-obesity group and obesity group calculated using Mann-Whitney U test and chi-squared test and Fisher’s exact probability test. *P* values are expressed as *P < 0.05 **P < 0.01 ***P < 0.001 ****P < 0.0001, versus Male, versus early elderly group, versus non-obesity group. early elderly group: 65 * łe *  Age < 75, late elderly group: 75  * łe *  Age < 90, non-obesity group: BMI < 25, obesity group: BMI * \ge *  25, smoker: number of current smokers, antihypertensive agents: number of patients taking antihypertensive agents

### ANOVA stratified by age and BMI for HOMA-IR values

An ANOVA stratified by age and BMI was conducted to examine the main effects of each factor on HOMA-IR values and the interaction effect between age and BMI. In men, the main effect of BMI was significant (F (1,254) = 26.67, *p* < 0.0001). However, in men, neither the main effect of age nor the interaction between age and BMI was statistically significant. BMI exerted a significant positive influence on HOMA-IR values in men (*p* < 0.0001). That is, in both groups aged 65 to 74 years and aged 75 to 89 years, obese men exhibited significantly higher HOMA-IR values than non-obese men (Fig. 1a).

In contrast, among women, neither the main effect of age nor that of BMI was statistically significant. However, the interaction between age and BMI was significant (F (1,216) = 4.76, *p* < 0.05). Given the significance of this interaction, in women aged between aged 65 and 74 years, BMI had a significant positive effect on HOMA-IR values (*p* < 0.05). Conversely, among women with a BMI of 25 or higher, age exerted a significant negative effect on HOMA-IR values (*p* < 0.05) (Fig. 1b).

Regarding HOMA-IR values, BMI alone exerted a significant effect in men, whereas in women, the combined influence of age and BMI was statistically significant.

### ANOVA stratified by age and BMI for HOMA-β values

A two-way ANOVA stratified by age and BMI was conducted to examine the main effects of each factor on HOMA-β values, as well as their interaction effect. In men, the main effect of age was not statistically significant, whereas the main effect of BMI (F (1,254) = 21.16, *P* < 0.0001) and the interaction between age and BMI (F (1,254) = 5.7, *P* < 0.05) were both statistically significant. In men, given the significant interaction effect, a simple main effects analysis was conducted. The results showed that, in men, BMI had a significant positive effect on HOMA-β values in both age groups (aged 65–74 years: *P* < 0.01; aged 75–89 years: *P* < 0.001).

Furthermore, among individuals with BMI * \ge * 25, age had a significant positive effect on HOMA-β values (*P* < 0.05). In other words, among men aged 65–74 and 75–89 years, those in the obese group had significantly higher HOMA-β values than those in the non-obese group (Fig. 1c).

In contrast, among women, neither the main effects of age and BMI nor their interaction were statistically significant.

These findings suggest that, in men but not in women, both BMI alone and the combined effect of age and BMI significantly influenced HOMA-β values (Fig. 1d).

### Logistic regression analysis of factors associated with elevated HOMA-IR values and reduced HOMA-β values

In the logistic regression analysis of factors associated with HOMA-IR values, higher BMI was significantly associated with increased HOMA-IR levels (*P* < 0.05). In the analysis of factors related to HOMA-β values, lower BMI and male sex were significantly associated with reduced HOMA-β values (*P* < 0.05 and *P* < 0.0001, respectively) (Table 2).

**Table 2:** Logistic regression analysis on variables affecting HOMA-IR and HOMA‐β

Variables	PE	OR	95%C.I.	*P*-value
*Variables affecting HOMA-IR * *≽2.5*				
Age(early elderly / late elderly)	− 0.041	0.960	0.175～5.267	N.S.
BMI(non-obesity /obesity)	− 1.361	0.256	0.078～0.844	*P* < 0.05
Interaction between age and BMI	− 0.525	0.591	0.064～5.434	N.S.
Sex (female/male)	− 0.972	0.378	0.126～1.135	N.S.
HbA1c (%)	− 1.467	0.231	0.039～1.376	N.S.
Antihypertensive agents	0.307	1.359	0.489～3.775	N.S.
Current smoker	0.340	1.406	0.170～11.608	N.S.
*Variables affecting HOMA-β < 30*				
Age (early elderly / late elderly)	− 0.322	0.725	0.243～2.159	N.S.
BMI (non-obesity /obesity)	0.788	2.198	1.189～4.063	*P* < 0.05
Interaction between age and BMI	0.476	1.610	0.478～5.422	N.S.
Sex (female/male)	− 0.840	0.432	0.284～0.657	*P* < 0.0001
HbA1c (%)	− 0.182	0.834	0.428～1.625	N.S.
Antihypertensive agents	0.086	1.090	0.724～1.641	N.S.
Current smoker	− 0.666	0.514	0.257～1.028	N.S.

PE, Parameter estimate; OR, Odds ratio; C.I., Confidence interval; early elderly group: 65  * łe * Age < 75; late elderly group: 75  * łe * Age < 90; non-obesity group: BMI < 25; obesity group: BMI  * \ge * 25; antihypertensive agents: patients taking antihypertensive agents

## Discussion

This study indicated that, among elderly men without a history of diabetes, increases in BMI were consistently accompanied by elevations in both HOMA-IR values and HOMA-β values.

A large-scale study of middle-aged Japanese individuals [16] also reported that, among participants with normal glucose metabolism confirmed by an oral glucose tolerance test (OGTT), a significant association between elevated HOMA-IR values and increased visceral fat was observed only in men. Similarly, in the present study, a significant increase in HOMA-IR values with increasing BMI was observed in both early and late elderly men. Furthermore, a large-scale study of non-diabetic Western men and women, primarily in their 30s and 40s [17], reported significant positive correlations between BMI and both HOMA-IR and HOMA-β values. In our study as well, similar associations were observed in early and late elderly men. In addition, a study using the euglycemic-hyperinsulinemic clamp method to examine sex differences in insulin resistance among obese elderly individuals [18] found that men exhibited higher insulin resistance than women, which supports the findings of the present study.

An increase in BMI is also due to an expansion of adipose tissue, which may induce insulin resistance through inflammatory cytokines associated with adiposity [19, 20]. The compensatory hyperinsulinemia observed in response to increased insulin resistance [21] is considered one of the factors contributing to elevated HOMA-β values.

Furthermore, the novelty of this study lies in the sex differences in HOMA-IR and HOMA-β values observed among obese elderly individuals. In particular, among late elderly women, the obese group exhibited lower HOMA-IR values compared to the non-obese group, while HOMA-β values were comparable between the two groups.

It has been reported that insulin resistance in women is more closely associated with visceral fat than with subcutaneous fat, as demonstrated in studies using the euglycemic-hyperinsulinemic clamp method [22] and in research targeting middle-aged Japanese women [23]. In contrast, a study using MRI to assess abdominal fat distribution [24] has shown that the amount of subcutaneous fat is significantly greater in women than in men. Moreover, a large-scale study of healthy, non-diabetic Japanese individuals [25] reported that, while subcutaneous fat significantly decreases with age in obese men, it remains relatively unchanged in obese women. This finding suggests that the proportion of subcutaneous fat tends to be maintained in elderly obese women despite advancing age. Although the present study did not quantify visceral and subcutaneous fat volumes, the finding that HOMA-IR values were lower in the obese group compared to the non-obese group among elderly women may be explained by the preservation of subcutaneous fat in the obese group.

On the other hand, a large-scale Japanese study comparing HOMA-β values [26] found that both younger adults aged 22–29 and middle-aged individuals aged 50 and older had higher HOMA-β values in women than in men, suggesting greater insulin secretory capacity in women. In our study as well, among late elderly women, despite lower HOMA-IR values in the obese group, HOMA-β values remained comparable to those in the non-obese group. This may reflect the inherently higher insulin secretory capacity in women.

The finding that HOMA-β values were comparable between elderly obese and non-obese women may be explained by several factors. A predominance of subcutaneous fat distribution may reduce the burden on pancreatic β-cells [27]. Moreover, muscle mass declines in elderly individuals with frailty [28] or sarcopenia [29] and women generally have less muscle mass than men [30]. Therefore, the relative age-related decline in muscle mass is smaller, which may result in more modest compensatory changes in insulin secretion in response to alterations in insulin resistance. In addition, estrogen exerts protective effects on pancreatic β-cells [31, 32], and hepatic insulin clearance [33] is lower in women than in men [34]. These factors may collectively contribute to the observed findings.

This study has several limitations. First, in this study, HOMA-β values were calculated based on FPG levels and F-IRI levels. Therefore, it is considered to reflect basal insulin secretion influenced by compensatory hyperinsulinemia and alterations in insulin clearance, rather than intrinsic β-cell function. In this regard, the present study was not able to directly assess intrinsic β-cell function. Second, considering that individuals with HbA1c levels between 5.8% and 6.5% are at increased risk of developing diabetes [35], the absence of an OGTT raises the possibility that the study population with a mean HbA1c (NGSP) levels of 5.8 ± 0.2% may have included individuals with borderline diabetes or undiagnosed diabetes who were not receiving treatment. Third, only BMI was measured in this study, while waist circumference was not assessed. As a result, it was not possible to evaluate visceral fat volume, which is closely associated with insulin resistance in obese individuals.

In conclusion, this study revealed marked sex-based differences in the trajectories of insulin resistance and basal insulin secretion across age and BMI subgroups among elderly individuals without a history of diabetes. These findings may provide valuable insights for understanding the pathophysiology of diabetes in the aging population and for selecting appropriate pharmacological interventions as elderly diabetic patients, whose numbers are expected to increase. Specifically, in elderly men, insulin resistance closely associated with obesity was firmly present regardless of age, whereas, in elderly women, there is no difference in basal insulin secretion between the obese and non-obese groups irrespective of age.

In future preventive medicine in the elderly, it will be essential to develop sex-specific strategies to prevent the onset of diabetes, taking into account gender differences in insulin dynamics associated with categories in BMI.
